# Video analysis of optic-haptic-interaction during hydrophobic acrylic intraocular lens implantation using preloaded injectors

**DOI:** 10.1186/s12886-023-03216-5

**Published:** 2023-12-19

**Authors:** Weijia Yan, Andreas F Borkenstein, Ramin Khoramnia, Eva-Maria Borkenstein, Gerd U Auffarth

**Affiliations:** 1 The David J Apple International Laboratory for Ocular Pathology, Department of Ophthalmology, University Hospital Heidelberg, Im Neuenheimer Feld 400, 69120 Heidelberg, Germany; 2 Borkenstein and Borkenstein, Private Practice at Privatklinik Kreuzschwestern, Graz, Austria

**Keywords:** Intraocular lens, Preloaded injector, Optic-haptic-interaction

## Abstract

**Objective:**

To compare the optic-haptic interaction of different hydrophobic acrylic IOLs after using six preloaded injectors.

**Methods:**

We reviewed the video-recordings of procedures on a total of 388 eyes that underwent phacoemulsification and intraocular lens (IOL) implantation. For six preloaded injectors: multiSert (Hoya Surgical Optics) [System 1], TECNIS Simplicity (Johnson & Johnson Vision) [System 2], TECNIS iTec (Johnson & Johnson Vision) [System 3], AutonoMe (Alcon, Laboratories) [System 4], Bluesert (Carl Zeiss Meditec) [System 5], and Prosert (OphthalmoPro GmbH) [System 6], we noted in each case the time of IOL delivery and made a descriptive observation of IOL insertion and optic-haptic-interaction.

**Results:**

We defined standard haptic behavior where the haptics emerged “folded” from the injector and quickly recovered their pre-implantation appearance. The incidence where the leading haptic emerged in a deformed way for System 1 was 20%, System 2: 19%, System 3: 14%, System 4: 56%, System 5: 24% and System 6: 5%. For trailing haptic deformed behavior, the incidence was 36%, 6%, 4%, 8%, 18% and 2%, respectively for Systems 1 to 6. Optic-haptic adhesion occurred in 2% of cases for System 1, 44% for System 2, 52% for System 3, 48% for System 4, and 11% for System 6 (P < 0.05). Adhesion was not found with System 5.

**Conclusions:**

We observed different deformed behavior for leading and trailing haptics in the six preloaded systems, some systems had as much as 52% optic-haptic adhesion.

## Introduction

Cataract surgery has witnessed a continuous stream of technological innovations in recent decades. A pivotal element in ensuring the safety and efficacy of such surgical procedures entails the precise placement of an intraocular lens (IOL). Within this context, the injector apparatus, its operational characteristics, and the properties of the IOL itself assume particular significance. The advancement of cataract surgery has facilitated the emergence of preloaded IOL delivery systems [1]. The move towards preloading injectors was accompanied by improvements in injector design, eliminating the necessity for manual loading of the IOL during the surgical procedure. Consequently, this has reduced procedural variability during IOL preparation and loading, ultimately contributing to a reduction in the overall duration of the surgical procedure [2–4].

There are, in general, two broad categories of preloaded injectors: push “syringe-style” injectors and screw injectors [5]. A recent addition to this classification involves an innovative injector system utilizing CO_2_ gas pressure for lens propulsion [5]. These categories diverge in cartridge and plunger design, injector system mechanics, and their respective interactions with the preloaded IOL, resulting in distinctive implantation behaviors associated with each preloaded IOL [6]. It is worth noting that preloaded IOL delivery systems have encountered challenges regarding predictability and safety [7–10]. In a study by Ong et al., only 45% of 85 cases exhibited accurate IOL delivery, necessitating additional rotational adjustments for the remaining 55% [7]. Furthermore, other problems associated with the preloaded IOL delivery system were reported, including adhesion between the haptic and the optic and inter-haptic adhesion. This phenomenon was particularly prevalent in single-piece IOLs [9–12]. While previous research has yet to address these adhesion issues extensively, their occurrence should be considered, and they may become more conspicuous, particularly with the introduction of new IOL models and injector systems. Therefore, we compared the lens delivery performances of six preloaded hydrophobic-acrylic IOL delivery systems. This analysis was conducted through a comprehensive review of recorded surgical procedures for routine cataract cases. In each case, we assessed the deformed presentation of two haptics, optic-haptic-interaction, and recorded the implantation time for placing the lens into the capsular bag.

## Materials and methods

### Study design and participants

Our study was an observational, retrospective study conducted at the eye clinic of Heidelberg University in Germany and a private practice, Privatklinik der Kreuzschwestern (Borkenstein & Borkenstein) in Graz, Austria. The Ethics Committees of the University of Heidelberg and Medical University Graz approved the protocol. All patients provided written informed consent, and the study followed the principles of the Declaration of Helsinki. We excluded patients requiring an intraocular lens outside the commercially available spherical power range. Critical exclusion criteria applied to each eye included pupil abnormality, corneal opacity, glaucoma, retina disease, and a history of ocular trauma or ocular surgery that was not resolved or stable.

### The preloaded IOL delivery systems

**Fig. 1:**
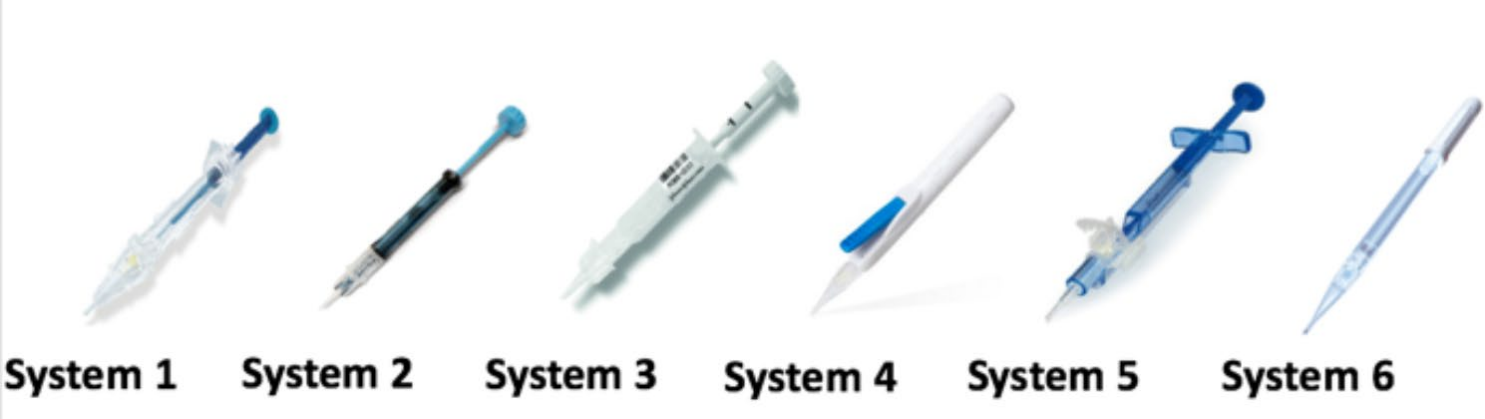
Images of six different injector systems

We examined video recordings of surgery using six different IOLinjectors, all preloaded with the IOL by the manufacturer at the IOL manufacturing site. All are single-use injectors that claim to promote more efficient OR procedural time (avoiding the lens loading step in the OR) and improved safety since the possibility of contamination of the IOL during a loading procedure in the OR is avoided (Table 1**and** Fig. 1**)**.

#### System 1

the multiSert (Hoya Surgical Optics, Singapore) has dual-insertion functionality that allows a choice between a two-handed screw or a one-handed syringe-style plunger enabling the surgeon to alter technique based on need or preference. It also has an adjustable insert shield for precise depth management of the injector tip.

***System 2***: TECNIS Simplicity and ***System 3***: the TECNIS iTec (both from Johnson & Johnson Vision, New Jersey, USA) are screw-style injectors for implantation of the TECNIS IOL into the capsular bag. The TECNIS Simplicity is the newer-generation version that allows either ophthalmic viscosurgical device (OVD) or balanced salt solution (BSS) fill within the injector to lubricate lens movement in the injector nozzle.

#### System 4

the AutonoMe (Alcon Laboratories, Inc., Fort Worth, TX, USA) is a CO_2_-gas-powered, automated, fully preloaded system for a one-handed lens advancement using a “speed controller’ to implant the Clareon IOL.

#### System 5

The Bluesert (Carl Zeiss Meditec, Jena, Germany) is loaded with the CT LUCIA 621P. It features a silicone plunger that avoids damage to the IOL and advances with less force than needed.

#### System 6

The Prosert (OphthalmoPro GmbH, Sankt Ingbert, Germany) is a screw injector with a precision screw thread designed to facilitate a one-step “into the bag” implantation process. Additionally, its dynamic tip permits implantations with incisions ranging from 2.0 to 2.2 mm, and the injector tip boasts an outer diameter of 1.78 mm for precision.

**Table 1:** Characteristics of the preloaded delivery systems

Parameter	System 1	System 2	System 3	System 4	System 5	System 6
**Delivery system**	multiSert (Hoya)	TECNIS Simplicity(AMO)	TECNIS iTEC (Johnson & Johnson)	AutonoMe (Alcon)	Bluesert (Zeiss)	Prosert (OphthalmoPro)
Driving mechanism	Screw type/ Pushed type	Screw type	Screw type	CO_2_ Propulsion type	Pushed type	Screw type
Implantation technique	two-handed/ single-handed manual advance	two-handed manual advance	two-handed manual advance	Single-hand automated advance	Single-hand manual advance	two-handed manual advance
Incision size (mm)	2.2	2.2 to 2.4	2.2 to 2.4	2.2	2.2 to 2.6	2.0 to 2.2
**Intraocular lens**						
Model	Vivinex XY1-SP	TECNIS DIB00	TECNIS PCB00	Clareon CNA0T0	CT LUCIA 621P	Primus-HD
Power (D)	+ 6.0 to + 30.0	+ 5.0 to + 34.0	+ 5.0 to + 34.0	+ 6.0 to + 30.0	+ 0.0 to + 34.0	-10.0 to + 36.0
Material	Hydrophobic acrylic	Hydrophobic acrylic	Hydrophobic acrylic	Hydrophobic acrylic	Hydrophobic acrylic	Hydrophobic acrylic
Design	1-piece, monofocal	1-piece, monofocal	1-piece, monofocal	1-piece, monofocal	1-piece, monofocal	1-piece, monofocal

### Surgical procedure

All surgeries were performed by three experienced surgeons (AFB, RK, and GUA) under local or general anesthesia. A Centurion (Alcon, USA) or WhiteStar Signature (Johnson & Johnson, USA) phacoemulsification machine and a 0.9 mm 45° ABS Intrepid Balanced phaco tip with a NanoSleeve (Alcon, Fort Worth, USA) were used to allow micro-coaxial phacoemulsification at the intended incision sizes without wound size enlargement. All the side-port incision was made with a 1.0 mm paracentesis knife and the main corneal incision was placed at the superior (12 o’clock) quadrant of the cornea near the limbus. Knife sizes were 2.2 mm (System 1, 2, 3, 4, and 6) or 2.4 mm (System 5), according to the recommendations provided by the manufacturer of each delivery system. The IOL was inserted into the capsular bag using the preloaded delivery system prepared by a trained nurse or surgical assistant. The same ophthalmic viscosurgical device (OVD) (Hydroxypropylmethylcellulose [20.0 mg]) at room temperature (20–23° C) was used in all cases. Then, surgeons depressed or screwed the plunger to introduce the IOL through the injector nozzle into the capsular bag.

### Video observation and data collection

Two authors (WJY and AFB) observed all the video recordings.

The main observations were: (1) deformed presentation of leading and trailing haptics, (2) optic-haptic-interaction during IOL delivery, (3) the time of each implantation step defined in seconds, calculated by adding the nozzle insertion and IOL unfolding together. Unfolding time is required for the folded IOL to recover 95% or more of its overall diameter before folding. The number of cases requiring additional manipulation with Sinskey hook, Irrigation/Aspiration (I/A) handpiece, and spatula to release the haptic adhesion was recorded. Other noncritical delivery events, including entrapment of trailing haptic, overriding of the syringe plunger over the optic, and damage to the optic edge, were also noted.

### Statistical analysis

All data were recorded into an Excel spreadsheet (Microsoft Corp), from which the mean and standard deviation were calculated for each measurement. The Shapiro-Wilk test confirmed the normal distribution of the data. To statistically compare the data from the eyes of each group, one-way analysis of variance (ANOVA) with Bonferroni adjustment for post-hoc comparisons was used to compare IOL delivery and IOL power among these six groups. Statistical analysis was carried out using GraphPad (Graphpad Software, US), and a *P* value less than 0.05 was chosen for statistical significance.

## Results

We enrolled 388 eyes of 367 patients (176 males, 212 females) in the study. 194 eyes (50%) underwent surgery by a surgeon (GUA or RK) at the eye clinic at Heidelberg University. The other 194 eyes underwent surgery in Private Practice Borkenstein & Borkenstein by one surgeon (AFB). The between-group difference in age, gender, and IOL power were not statistically significant (*P* > 0.05). The baseline demographics of

the study population are summarized in Table 2.

**Table 2:** Patient demographics and delivery characteristics

Characteristics	System 1	System 2	System 3	System 4	System 5	System 6
**No. of eyes**	50	82	99	63	50	44
**Age (yr)**	72.7 ± 7.8	67.7 ± 8.5	68.6 ± 12.6	71.3 ± 7.8	73.8 ± 6.7	70.9 ± 6.5
**M/F**	22/28	38/44	45/54	33/30	20/30	18/26
**OD/OS**	29/21	47/35	48/51	32/31	27/23	19/25
**IOL power (D)**	22.0 ± 2.5	20.9 ± 4.2	21.1 ± 3.8	20.9 ± 3.6	21.8 ± 2.8	22.1 ± 2.7
**IOL delivery time (s)**	19.9 ± 6.5	28.5 ± 5.6	34.8 ± 9.8	23.8 ± 7.3	22.2 ± 2.6	16.6 ± 4.1

OD: oculus dexter; OS: oculus sinister

### Presentation of leading and trailing haptic during the insertion

**Fig. 2:**
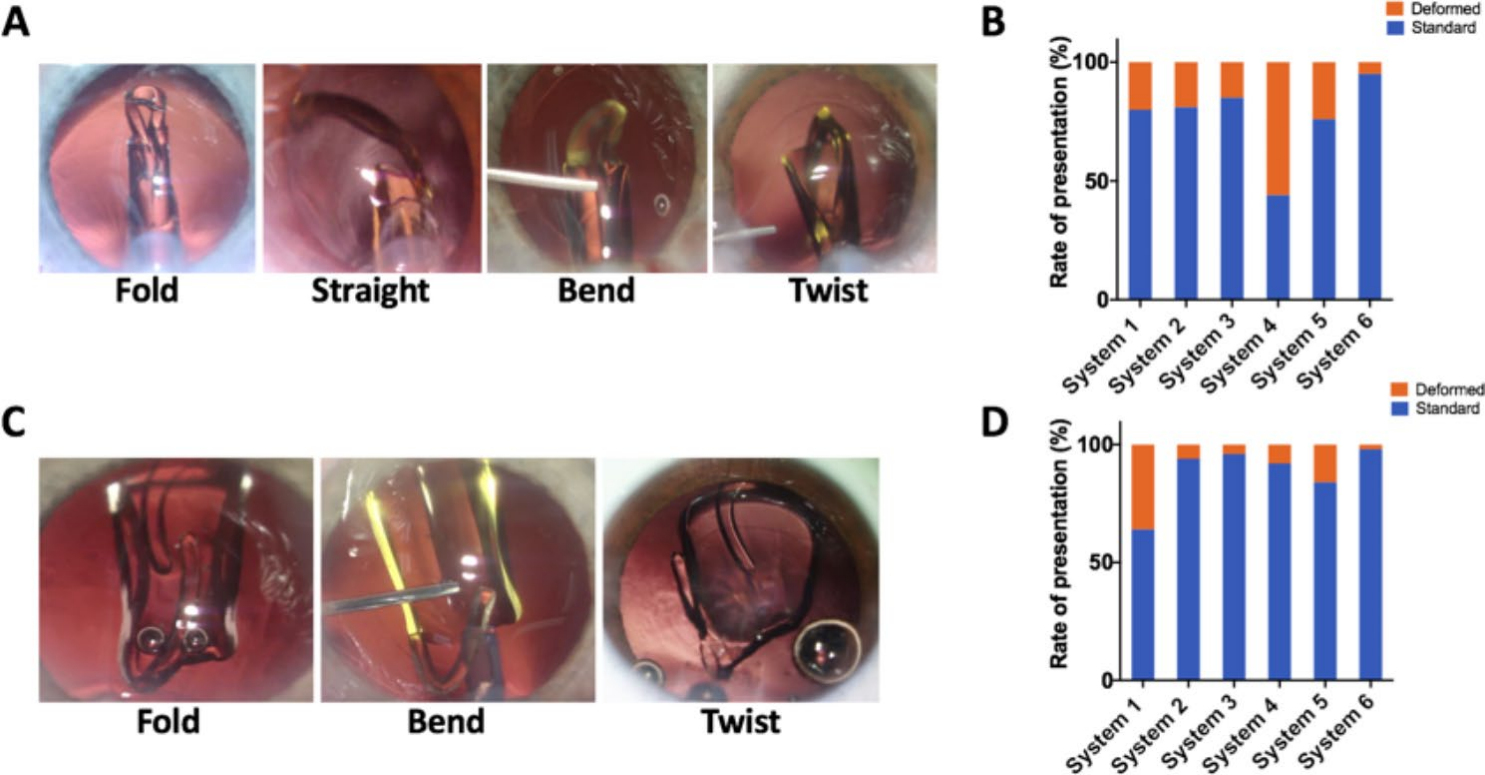
Presentation of leading (**A**) and trailing (**B**) haptics. (From left to right: Fold, Straight, Bend and Twist). Standard and deformed rate of haptic presentation for leading and trailing haptics (**B** and **D**)

The standard performance expectation for haptics is to exhibit a “fold” during delivery. However, deviations from this ideal behavior can occur, resulting in what we classify as “straight”, “bend” and “twist” delivery behaviors when the leading or trailing haptic enters the capsular bag. The haptics exhibit a “Straight” behavior when they maintain a straight orientation along the barrel of the cartridge as they move forward within the cartridge nozzle; “Bend” occurs when the haptics fold incompletely as they travel within the cartridge nozzle or remain partially folded beneath the optic during release; “Twist” behavior indicates an incorrect orientation that requiring a 180-degree injector rotation to position the haptic properly (Fig. 2A **and C**).

System 1 experienced ten instances of deformed delivery behavior in the leading haptic (20%) and eighteen in the trailing haptics (36%). In System 2, there were sixteen occurrences of deformed delivery behavior in the leading haptic (19.5%) and five in the trailing haptics (6.1%). System 3 had fourteen cases of deformed delivery behavior in the leading haptic (14.1%) and five in trailing haptics (4%). In System 4, thirty-five cases displayed deformed delivery behavior in the leading haptic (55.6%), and five cases showed it in the trailing haptics (7.9%). System 5 recorded twelve cases of deformed delivery behavior in the leading haptic (24%) and nine in trailing haptics (18%). System 6 presented two cases of bend performance (4.5%) classified as deformed delivery behavior for the leading haptics and one case of bend performance (2.3%) for the trailing haptics, which we also considered as deformed delivery behavior **(**Fig. 2**BD and** Table 3**).**

**Table 3:** Incidence of leading and trailing haptic presentation in six systems

	Leading Haptic				Trailing Haptic		
	Standard	Deformed			Standard	Deformed	
	Fold	Bend	Twist	Straight	Fold	Bend	Twist
System 1	40(80%)	7 (14%)	2(4%)	2(2%)	32(64%)	18(36%)	0
System 2	66(81%)	14(17%)	1(1%)	1(1%)	77(93.9%)	3(3.7%)	2(2.4%)
System 3	85(85%)	10(10%)	2(2%)	2(2%)	94(95%)	2(2%)	3(3%)
System 4	28(44%)	18(29%)	15(24%)	2(3%)	58(92%)	1(1.6%)	4(6.3%)
System 5	38(76%)	4(8%)	8(16%)	0	41(82%)	9(18%)	0
System 6	42(95%)	0	2(5%)	0	42(95%)	2(5%)	0

### Optic-haptic adhesion

**Fig. 3:**
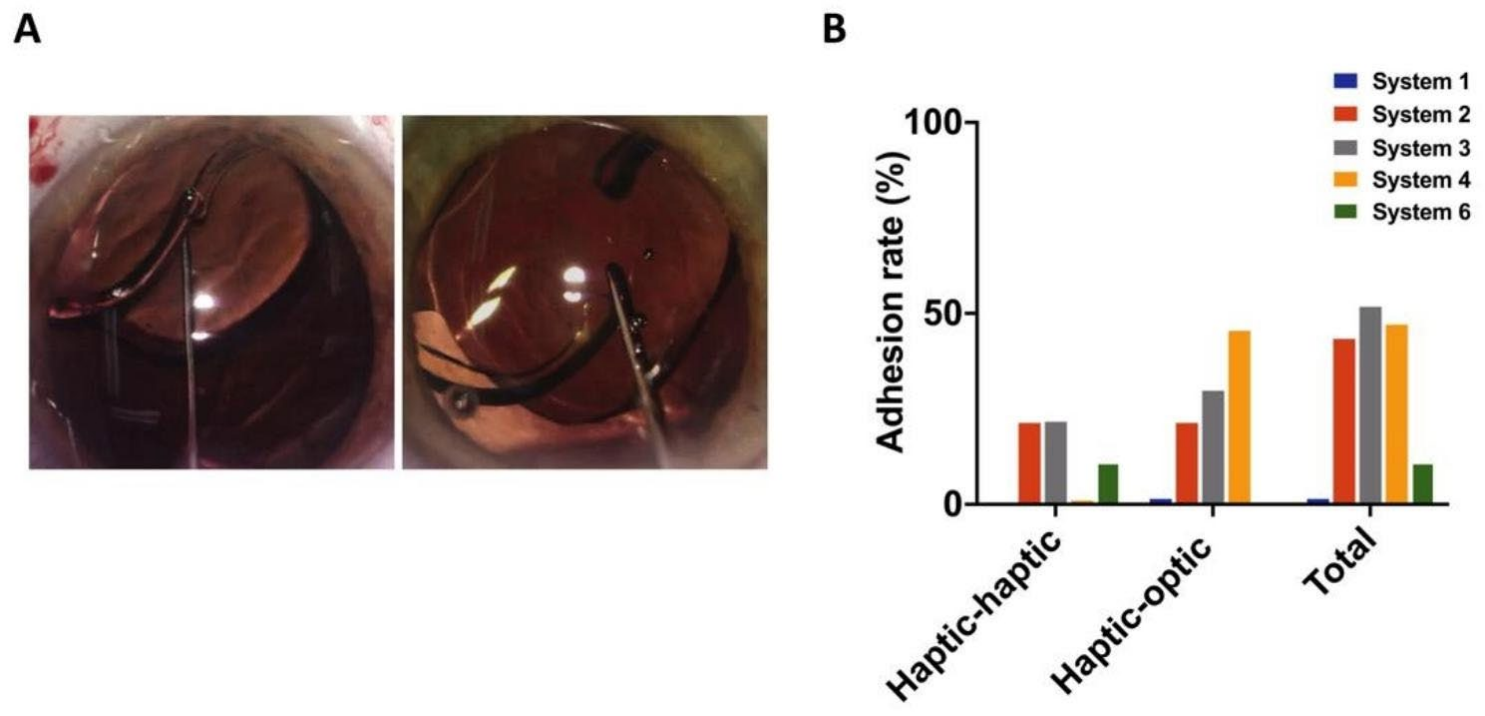
Adhesion Scenarios. Presentation of haptic-haptics adhesion (Left) and haptic-optic adhesion (Right) (**A**). Adhesion rate between the two haptics and between haptic and optic (**B**)

Adhesion occurred in Systems 1, 2, 3, 4, and 6 (Fig. 3A). We did not observe Adhesion in System 5. System 1 did not show any bonding between the haptics but had one case (2%) of adhesion between the haptic and optic. Thirty-six cases (43.9%) of adhesion occurred in System 2, where half (21.9%) was adhesion between two haptics and half was haptic-optic adhesion (21.9%). System 3 had fifty-two cases (52.5%) of adhesions, twenty-two cases (22.2%) between two haptics, and thirty cases (30.3%) between haptic and optic. System 4 had thirty cases (47.6%) of adhesion, with the majority (46%) occurring between haptic and optic. System 6 had five cases (11.3%) of adhesion, all haptic-to-haptic adhesion (Fig. 3B).

### Implantation time

**Fig. 4:**
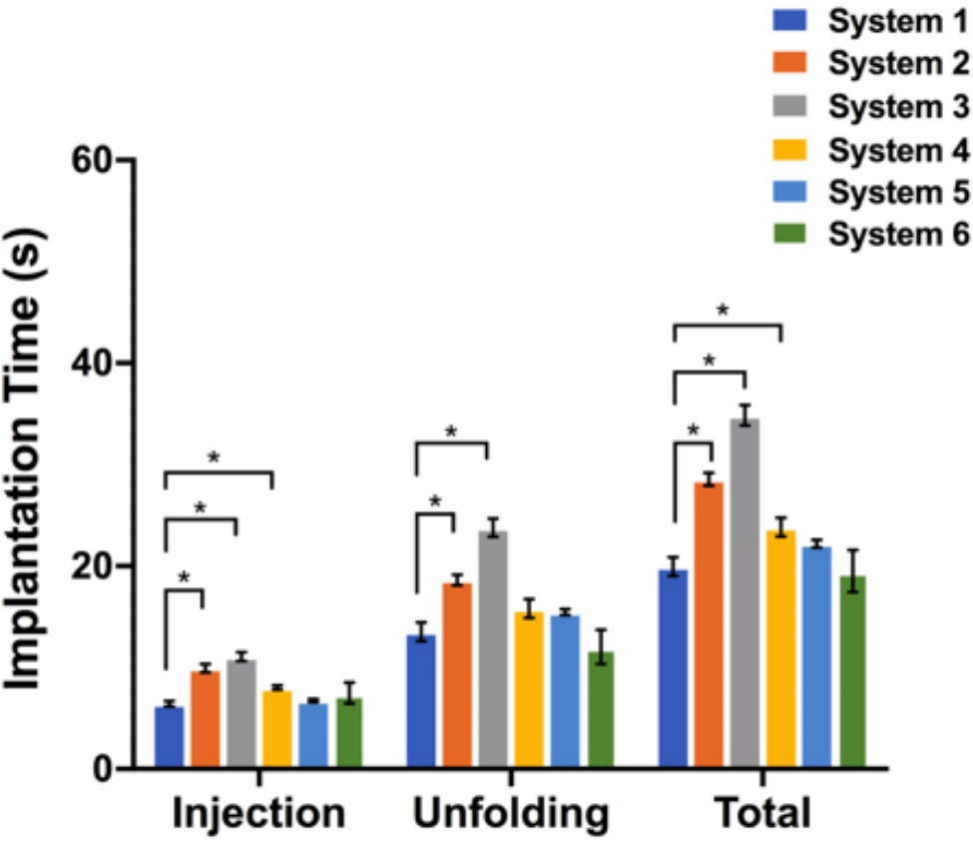
Total mean implantation time and mean implantation time of each phase for each delivery system. * *P* < 0.05

Figure 4 showed the results of the total implantation time. System 3 showed the longest mean delivery time (34.8 ± 9.8), whereas System 6 had the shortest (16.6 ± 4.1). Most systems had significant differences in mean total implantation time (*P* < 0.05). No significant differences were showed between Systems 1 and System 5, System 1 and System 6 **(**Fig. 4**)**.

### Additional manipulation

The number of cases requiring additional manipulation to release the adhesion and perform rotational adjustments to optimize the IOL’s orientation within the central of the capsular bag was: 1/50 (2%) for System 1, 23/82 (28.1%) for System 2, 32/99 (32.3%) for System 3, 32/63 (50.8%) for System 4, 12/50(24%) for System 5, and 2/44 (4.5%) for System 6.

### IOL power

**Fig. 5:**
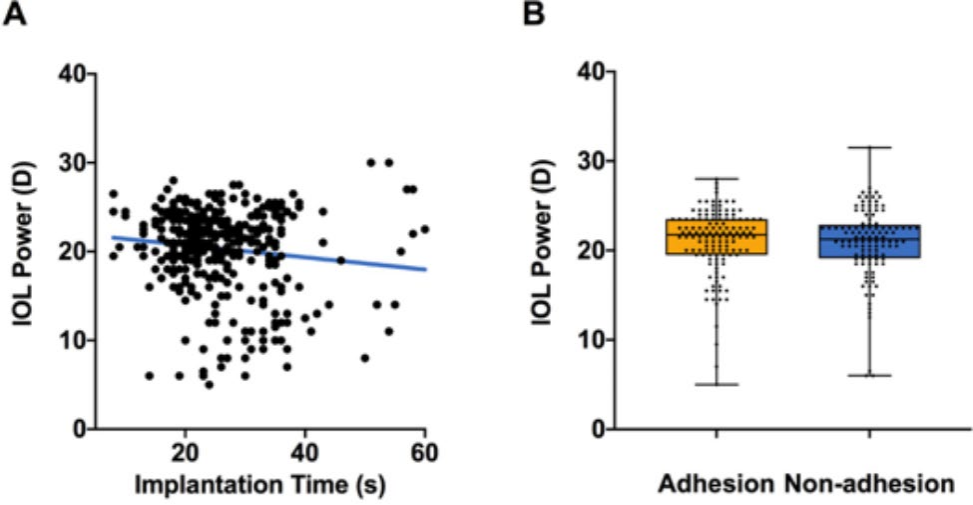
The correlation between IOL power and implantation time (**A**), occurrence of adhesion (**B**)

The IOL power exhibited a statistically significant difference in implantation time (P < 0.0087). Nonetheless, no significant differences were observed regarding adhesion occurrences between the two haptics or between haptic and optic **(**Fig. 5**).**

### Noncritical delivery events

No cases in any systems showed the situation of entrapment of trailing haptic, overriding of the syringe plunger over the optic, and damage to the optic edge.

## Discussion

We compared six preloaded injector systems by reviewing video-recorded routine cataract surgery. We took note of the presentation of leading and trailing haptics, optic-haptic-interaction, and lens delivery time. The six systems have various designs, are made of different materials, and are loaded with different lens models, all leading to patterns of lens unfolding behavior. The adhesion of the haptic and optic surfaces has been described as the “handshake phenomenon”. The strength of adhesion appears to vary according to the IOL material. Overall, System 3 has the highest adhesion rate and longest implantation time compared to the other systems.

Numerous studies have indicated that the preloaded system provides safety, reproducibility, and effectiveness during the IOL implantation [10, 11, 13]. Khoramnia et al. evaluated AutonoMe (Alcon) and iSert (Hoya) preloaded systems using video-based analysis, revealing a noteworthy absence of potentially critical events during surgery, which may be attributed to the advanced design of preloaded injectors [10]. Borkenstein et al. also examined the safety aspects of preloaded compared with non-preloaded systems, which shows that preloaded systems streamlined and enhanced the safety of IOL implantation, reducing the risk of IOL damage [13]. In regards of safety, our study did not reveal any critical complication during IOL delivery, with only minor implantation incidents observed. Most occurrences involved harmless haptic-optic adhesions or deformed presentations of the leading or trailing haptic. These resolved spontaneously or were addressed through minor adjustments by the surgeon using additional instruments. Zhang et al. compared the delivery performance of 4 injector systems in porcine cadaver eyes, including multiSert (Hoya), UltraSert (Alcon), iTec (Johnson & Johnson Vision), and RayOne (Rayner), through Miyake-Apple view videos [11]. Regarding abnormal leading haptic behaviour, they observed a single case (10%) for both multiSert and UltraSert and two cases (20%) of RayOne. However, we exhibited a higher occurrence of deformed leading haptic behavior, with 20% for multiSert and 14% for iTec. This variance may be attributed to differences in the criteria used to define deformed haptic behavior between the studies. Although these deformed haptic behaviour can occur during the insertion, they do not generally affect the IOL implantation process or cause additional damage.

Several factors contribute to the observed deformations in both leading and trailing haptic behavior during lens implantation. A recent study by Borkenstein. used computed tomography to compare the different geometric features of the optic-haptic junction (OHJ), haptic thickness and area, and OHJ volume and surface-area of five hydrophobic IOLs. The collected evidence refuted opinions that all hydrophobic acrylic one-piece IOLs with the same overall diameter are alike. The authors concluded that lens models could have considerable differences in geometry affecting the lens behavior in different capsular bag sizes [14]. The results may align well with ours, as lenses with thick OHJ and haptics unfold differently than lenses with thin OHJ when inserted through the same-sized continuous curvilinear capsulorhexis (CCC). This could explain why some lenses have specific unfolding behavior. However, to attain an objective evaluation, it is important to assess the consistency of the unfolding behavior. In our investigation, all six injector systems demonstrated notable reproducibility, with the leading and trailing haptics being predominantly introduced into the capsular bag in a standard behavior (Table 3). However, in the case of System 4, only 44.4% of the leading haptics exhibited the expected delivery performance. Deformed haptics, in particular, amplified the likelihood of interactions with the optical surface, subsequently giving rise to adhesions, some of which necessitated additional instrumental manipulation or repositioning, thereby augmenting the surgical risks. Our study showed that System 4 exhibited the second-highest occurrence of adhesion among the six systems (47.6%), with most of these cases involving interactions between the haptic and optic components. Therefore, the highest proportion requires additional surgical instruments. Consequently, reproducibility significantly contributes to safety within the operating room, allowing surgeons to concentrate on standardized procedures and minimize unforeseen complications.

Complications can occur when the IOL does not fully unfold in the bag [15]. Iwase and Tanaka reported that incomplete unfolding of the hydrophobic acrylic Hoya AF-I caused intraoperative rotation of the IOL in the capsular bag. The tendency of the unfolding and rotation can result in optical rotation and potential contact with the endothelium or inverted implantation (the implant positioned upside down) [16, 17]. Decreased implantation time could reduce the risk of complications arising from incomplete IOL unfolding. Khoramnia et al. compared the quality and duration of two preloaded injectors (AutonoMe and iSert). The implantation time included docking, injection, and unfolding [10]. Our study’s the overall implantation time included the nozzle insertion and the IOL unfolding phase. However, the surgeon could proceed with parallel surgery steps (irrigation/aspiration), and the unfolding time should not be clinically relevant. Therefore, the injection time of our study was 6.4 ± 1.9 s for System 1 and 8.0 ± 1.6 s for System 4, which showed a slightly shorter but comparable injection time compared with the study of Khoramnia et al. (8.6 ± 1.9 and 10.0 ± 2.1s). Additionally, Chung et al. comparing the performance of 5 injectors found that the IOL delivery time ranged from 14.6 ± 2.1 s with the preloaded Tecnis PCB00 to 43.2 ± 6.5 s with the manually loaded EnVista MX60 [18]. Our study only used preloaded system, showing a shorter implantation time with six different preloaded systems compared to the manually loaded injector system. Therefore, we can confirm that preloaded injectors generally reduce the time needed for IOL implantation, thereby reducing the possibility of complications.

In the past, when lenses were loaded in injectors in the OR, measures were taken to avoid unpredictable unfolding: BSS was used instead of OVD, the IOL was wetted before being placed in the injector, care was taken to align the lens within the cartridge of the injector, or OVD had to be used that had a particular temperature [19, 20]. However, the current view is that the handshake phenomenon depends on material stickiness; some haptics are slightly sticky and soluble on their own or after rinsing. In some cases, the IOL needs a secondary instrument and through-the-incision manipulation of the nozzle to release the adhesion or to aid the IOL’s centration. This additional manipulation is a recognized hazard due to the increased risk of damage to the IOL, the iris, and the ocular structure (lens capsule). It also adds to increased surgical time [18]. Acar et al. reported that ocular surface bacteria contaminate the aqueous humor in 7–43% of cataract operations [21]. Every IOL and surgical fluid in contact with a surgical instrument increases the risk of bacterial contamination from the surgical field, which is not entirely sterile [21]. In our study, System 4 (50.8%) had the highest rate requiring manipulation to release the optic-haptic adhesion during the IOL implantation. This observation is slightly better than the study by Ong et al. on the AcrySert preloaded IOL delivery system (55% required additional manipulation) [7]. Although all cases in the present study did not have an adverse outcome, it could also decrease the operating room efficiency by increasing the time required for IOL loading, particularly in high-volume cataract practices. An ideal IOL injector system should have minimal or no unfolding problems during the implantation to avoid additional manipulation, shorten the entire operation, and reduce the risk of postoperative inflammation and infection.

One limitation of the study was the absence of measurements for resistance forces during IOL ejection, which impacts the IOL implantation process. Additionally, the lack of a control group (non-preloaded injectors) restricts the ability to compare techniques. Lastly, the study used the same type of OVD in all cases, but different OVDs should be evaluated in future studies to assess their impact on adhesion scenarios.

## Conclusion

In conclusion, the interaction of IOL and injector appears to be acceptable for six delivery systems. All systems showed a safe IOL implantation, and this confirmed past studies. Nevertheless, our data also emphasizes and indicates that there is still room for improving preloaded injectors, as no system shows an absence of undesired haptic behavior.

## Data Availability

All related data were displayed in the manuscript. Further information regarding the data can be obtained by contacting the corresponding authors.

## Abbreviations

CCC: Curvilinear capsulorhexis
IOL: Intraocular lens
IOP: Intraocular pressure
OHJ: Optic-haptic junction; OVD: ophthalmic viscosurgical device
